# Methodology Approach for Microplastics Isolation from Samples Containing Sucrose

**DOI:** 10.3390/molecules29173996

**Published:** 2024-08-23

**Authors:** Kornelia Kadac-Czapska, Beata Bochentyn, Aleksandra Maślarz, Sebastian Mahlik, Małgorzata Grembecka

**Affiliations:** 1 Department of Bromatology, Faculty of Pharmacy, Medical University of Gdańsk, 80-416 Gdańsk, Poland; kornelia.kadac@gumed.edu.pl (K.K.-C.); olamaslarz@gumed.edu.pl (A.M.); 2 Advanced Materials Center, Faculty of Applied Physics and Mathematics, Gdańsk University of Technology, 80-233 Gdańsk, Poland; beata.bochentyn@pg.edu.pl; 3 Institute of Experimental Physics, Faculty of Mathematics, Physics and Informatics, University of Gdańsk, 80-308 Gdańsk, Poland; sebastian.mahlik@ug.edu.pl

**Keywords:** microplastics, polyethylene, sucrose, isolation, pre-treatment, digestion, filtration, identification, scanning electron microscopy, µ-Raman spectroscopy

## Abstract

The growing production and use of plastics significantly contribute to microplastics (MPs) contamination in the environment. Humans are exposed to MPs primarily through the gastrointestinal route, as these particles are present in beverages and food, e.g., sugar. Effective isolation and identification of MPs from food is essential for their elimination. This study aimed to evaluate factors influencing the isolation of MPs from sucrose solutions to determine optimal conditions for the process. Polyethylene particles were used to test separation methods involving chemical digestion with acids and filtration through membrane filters made of nylon, mixed cellulose ester, and cellulose acetate with pore sizes of 0.8 and 10 µm. The effects of temperature and acid type and its concentration on plastic particles were examined using scanning electron microscopy and µ-Raman spectroscopy. The results indicate that increased temperature reduces solution viscosity and sucrose adherence to MPs’ particles, while higher acid concentrations accelerate sucrose hydrolysis. The optimal conditions for MPs’ isolation were found to be 5% HCl at 70 °C for 5 min, followed by filtration using an efficient membrane system. These conditions ensure a high recovery and fast filtration without altering MPs’ surface properties, providing a reliable basis for further analysis of MPs in food.

## 1. Introduction

Microplastics (MPs) are plastic particles ranging from 0.1 to 5000 μm, occurring in all colors or being transparent, with a regular or irregular shape, regardless of origin [1]. These fragments constitute a growing hazard to human health [1,2,3] because they can cause oxidative damage to lipids, genetic material degradation, activation of mitogen-activated protein kinase (MAPK) signaling pathways, breaches in cell membranes, impaired mitochondrial function, lysosomal malfunction, inflammatory responses, and the initiation of programmed cell death (apoptosis) [2]. Their widespread presence in aquatic and terrestrial environments exacerbates the issue [4], particularly through the contamination of food and beverages [3,5,6,7,8,9,10,11,12,13,14,15,16,17,18,19,20,21,22,23,24]. Thus, it is crucial to understand MPs’ presence in commonly consumed products, like sugar.

It has been discovered that MPs are present in sugar imported from both Asia and Europe. However, the extracted particles from German samples (>40 μm) were smaller than those from Bangladeshi samples (<300 μm) [9,23]. Moreover, polyethylene (PE), polypropylene (PP), poly(ethylene terephthalate) (PET), poly(acrylonitrile-co-butadiene-co-styrene) (ABS), poly(vinyl chloride) (PVC), poly(ethylene-co-vinyl acetate) (EVA), cellulose acetate (CA), polytetrafluoroethylene (PTFE), polycarbonate (PC), and nylon were identified in the analyzed samples [9,10,23]. Microplastics may be transported with contaminated sugar for beverages, nectars, energy drinks, soft drinks, and sweetened teas [10,11,24]. These particles might originate from packaging, textiles, and production processes (e.g., grinding, milling, internal transport, and filtration).

Developing an accurate and consistent research methodology is crucial for effectively investigating the presence of plastic particles in sucrose-containing products and understanding their potential risks to public health. The efficiency of the process can be significantly influenced by the choice of MP isolation method. This method has enabled the comparison of test results and providing a precise analysis of plastic particles.

The degradation of plastic particles during the isolation procedure could lead to incorrect conclusions about the quantity and properties of MPs. Therefore, to guarantee the precision and dependability of test results, it is crucial to create MP isolation techniques that reduce the likelihood of particle degradation. It should be noted that there are two primary steps in the process of separating MPs from food: digestion and filtration. The purpose of digestion is to remove the food matrix. The degradation of plastic particles should not be the outcome. There are studies concerning sugar with H_2_O_2_ as a digestion agent [23]. On the other hand, filtration—which concludes with the removal of plastic particles—should be characterized by the maximum MP recovery and occur as quickly as is feasible.

In our study, inorganic acids were used as digestion agents. In research on MPs, inorganic acids of different potencies are frequently applied to food and environmental samples to successfully remove the food matrix [25]. The two categories of inorganic acids that are most commonly used are oxyacids and anaerobic acids. Nitric(V) acid is an example of the first category. It is an oxidizing agent and a potent mineral acid. As such, HNO_3_ is well suited to the relatively rapid destruction of biogenic components. There are multiple protocols that use hot nitric acid treatment to aid in the digestion of flora and fauna [26,27,28,29]. Hydrochloric acid is an example of the second category. It is an aqueous solution of hydrogen chloride gas that has been used to digest zooplankton [30] and fish tissues [31].

In this paper, we report a methodological strategy for the efficient isolation of plastic particles from sucrose-containing samples without compromising their surface characteristics. Specifically, we examined whether the presence of sucrose has limited the isolation and identification of MPs. Moreover, we determined which separation techniques have proven to be the most useful for these samples, and assessed the influence of various physicochemical factors on the efficiency of the isolation process.

The extraction of MPs from sucrose solutions is an essential step in the assessment of food contamination by plastic particles for several reasons. Firstly, the development of an effective method for separating MPs allows for a detailed examination of their number and characteristics in product samples, such as beverages with a high sucrose content. It would be impossible to conduct a quantitative or qualitative analysis of MPs without effective isolation. Secondly, efficient separation of MPs is necessary to investigate their effects on live organisms. Accurately separating the particles from samples allows for experiments that assess the toxicity of plastic particles in quantities observed in food to be conducted. Thirdly, the isolation of MPs is necessary for both monitoring and assessing the extent to which the measures taken to reduce the amount of plastic particles contaminating food have been successful. The research described in this paper may help to standardize research procedures, enhance the consistency and reliability of results, and support efforts aimed at reducing the contamination of food products.

## 2. Results

Our research approach considered a number of variables that could affect the degree to which plastic particles (intentionally inserted PE with sizes between 34 and 50 µm) are isolated, including the test solution’s temperature (50, 60, or 70 °C), the type of filter being used (nylon filters with a pore size of 0.8 µm—N_0.8, nylon filters with a pore size of 10 µm—N_10, mixed cellulose ester filters with a pore size of 0.8 µm—MCE_0.8, mixed cellulose ester filters with a pore size of 10 µm—MCE_10, cellulose acetate filters with a pore size of 0.8 µm—CA_0.8, and cellulose acetate filters with a pore size of 10 µm—CA_10), and different digestive agents (HCl or HNO_3_ solutions at three concentrations of 1.9%, 3.5% or 5.0%). Systems without or with sucrose were compared.

### 2.1. Evaluation of Digestion Methods Using Scanning Electron Microscope

This study examined whether the temperature and chemical components used affected the degradation of plastic particles. Additionally, we focused on developing an isolation technique that can effectively separate MPs from sucrose solutions. It would not be advantageous for MPs to degrade during isolation from food matrices for a number of reasons. Firstly, the deterioration of plastics would change the original properties and structure of the particles, thereby affecting the test results. Secondly, the breakdown of MPs that results in their fragmentation may prevent the structural identification of polymers. Small fragments are harder to locate and examine; thus, studies on the contamination of food by such plastic particles might not be as reliable. Additional chemical alterations caused by degradation could hinder the interpretation of test results and lead to unclear or misleading conclusions.

**Figure 1:**
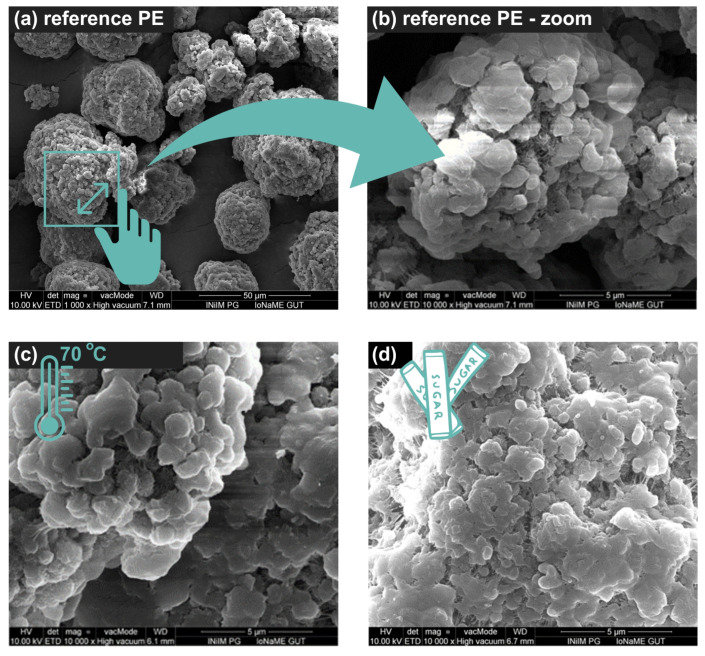
SEM images of (**a**) reference PE × 1000, (**b**) reference PE × 10,000, (**c**) PE treated at 70 °C, and (**d**) PE with sucrose treated at 70 °C.

The changes in the surface structure of the plastic particles were analyzed using scanning electron microscopy (SEM). The reference point for observing the changes was an image of a reference PE (“raw”) with a particle size of 34–50 µm, as shown in Figure 1a,b.

Temperature had no effect on PE degradation during the analyses that were conducted (Figure 1c). However, the amount of sucrose in the studied systems seemed to have an impact on the appearance of MPs (Figure 1d). Agglomerate formation was observed. Particle agglomeration was found to decrease with temperature.

**Figure 2:**
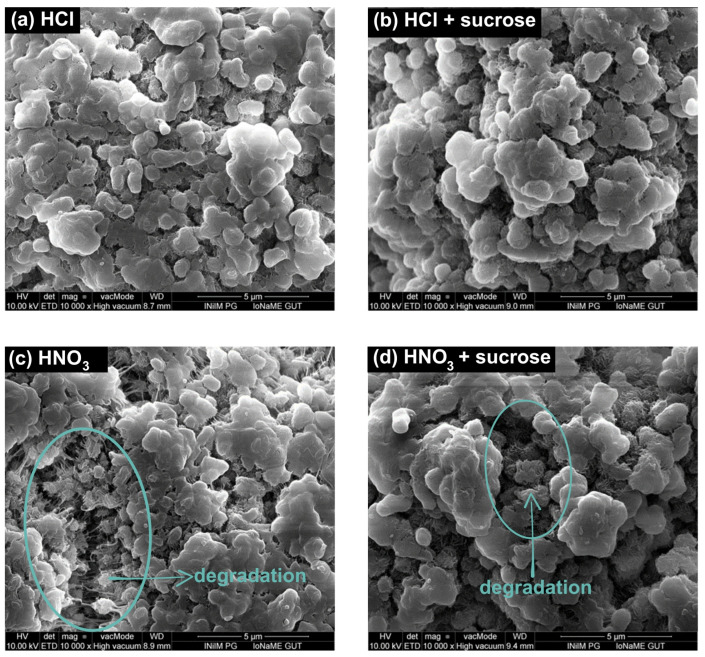
SEM images of (**a**) PE treated with 5% HCl, (**b**) PE with sucrose treated with 5% HCl, (**c**) PE treated with 5% HNO_3_, and (**d**) PE with sucrose treated with 5% HNO_3_.

Experiments with 1.9, 3.5, and 5.0% HCl solutions and MPs did not reveal any changes on the particles’ surfaces (Figure 2a). The interaction of HCl in solutions containing MPs and sucrose was also studied. Sucrose forming a coating/protective layer on the plastic particles, even at the lowest acid concentration (1.9%), underwent hydrolysis, which proceeded more efficiently at higher HCl concentrations. There was a deepening loss in the sucrose layer observed in 1.9% and 3.5% solutions. In the 5% HCl solutions, neither sucrose nor changes in the MPs’ surface relative to the reference were noted (Figure 2b).

In the case of testing solutions containing MPs and HNO_3_, the destruction of the PE surface was evident in HNO_3_ solutions with a concentration of 1.9% and progressed as the acid concentration increased. This process was manifested by an increased MP surface morphology (Figure 2c). The effect of HNO_3_ in solutions containing MPs and sucrose was also investigated (Figure 2d). As with the sucrose and HCl samples, the presence of sugar was evident in solutions with acid concentrations of 1.9% and 3.5%, respectively. Even the smallest concentration of acid used caused the hydrolysis of sucrose. The sucrose layer hydrolyzed, exposing the surface of the particles and allowing the acid to interact directly with the polymer. A 5% HNO_3_ solution increased the surface shape of MPs, which may have indicated a PE degradation process that was not observed in analogous systems with HCl.

### 2.2. Evaluation of Digestion Methods Using µ-Raman Spectroscopy

Polymer Raman spectra analysis is a valuable tool in the investigation of plastic degradation. The spectral analysis results for standard MPs and PE exposed to varying temperatures, as well as their reactions with sucrose and acids, are presented in this section.

**Figure 3:**
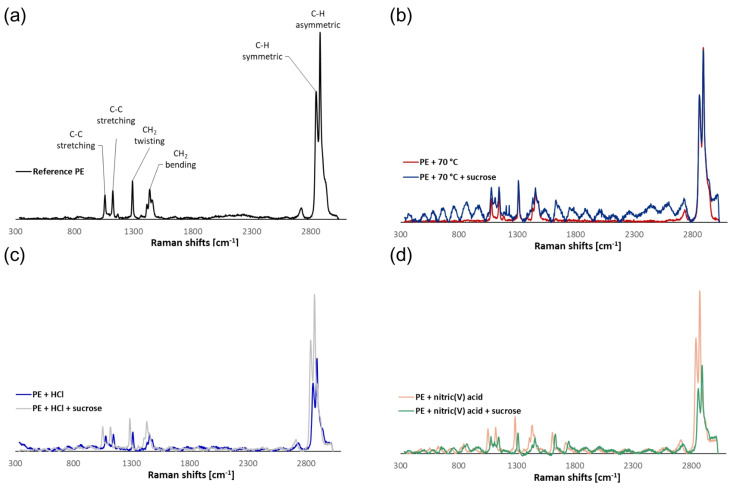
Examples of Raman spectra of (**a**) reference PE, (**b**) PE treated at 70 °C, (**c**) PE treated with 5% HCl, and (**d**) PE treated with 5% HNO_3_.

A comparison was made between the reference polymer’s Raman spectrum and the spectra obtained for particles treated at various temperatures (up to 70 °C) (Figure 3a,b).

It was discovered that temperature had no effect on surface structural changes. Unfortunately, undesired signals and “noise” were noticed during the testing of sucrose systems, indicating the presence of sugar on the MPs’ surfaces. This method yields extremely complex spectra, and assigning bands to specific vibrations is difficult. There were visible bands originating from sucrose molecules (1106 cm^−1^—C-O-H deformation; 1205 and 1230 cm^−1^—C-C-H, O-C-H, and C-O-H deformation). Owing to sucrose residues, it can be challenging to analyze MPs in actual food samples. This demonstrates the necessity of removing the food matrix from the products under analysis, which can be done by acid digestion.

No changes were observed on the PE surface when comparing the spectra of the reference polymer with those obtained for particles exposed to HCl at a temperature of 70 °C. This was confirmed by the results of the SEM analysis. Similarly, no new bands appeared in the sucrose-containing systems, which could indicate the breakdown of PE particles or that sucrose residues adhered to the MPs’ surfaces (Figure 3c).

The effect of HNO_3_ on the surface of MPs was confirmed by µ-Raman spectroscopy. New functional groups were visible in the Raman spectra of the PE that was undergoing surface degradation when exposed to a 5% HNO_3_ solution (Figure 3d). The formation of bands characteristic of C=O stretching vibrations at 1609 and 1713 cm^−1^ [32] was observed. In the case of systems with HNO_3_ and sucrose, the formation of analogous bands was confirmed at 1625 and 1739 cm^−1^. In addition, a band from C-O-H deformation vibrations appeared near 1086 cm^−1^, which is indicative of a sucrose residue. Changes in the intensity of the bands relative to the reference polymer were noticed due to the partial oxidation and dehydrogenation of the polymer, particularly with respect to C-H stretching vibrations in the range of 2800–3000 cm^−1^. In addition, the introduction of new functional groups and changes in the chemical structure of PE resulted in a shift of the bands characteristic of C-C stretching, CH_2_ twisting, and CH_2_ bending vibrations in the 1030–1490 cm^−1^ range.

### 2.3. Determination of Filtration Time and Recovery

**Figure A1:**
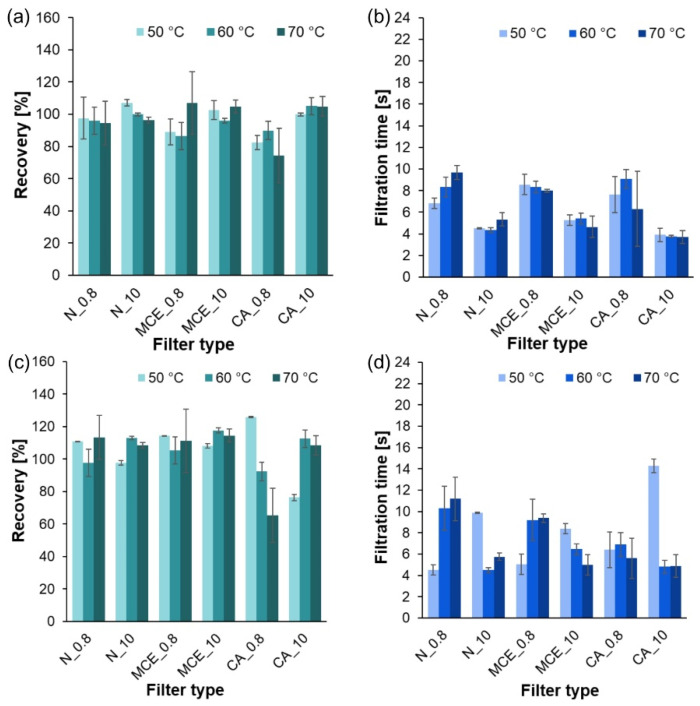
Results of the filtration process of systems subjected to temperatures of 50, 60, and 70 °C: (**a**) recovery of PE in the system without sucrose; (**b**) filtration time of the system without sucrose; (**c**) recovery of PE in the system with sucrose; (**d**) filtration time of the system with sucrose.

**Figure A2:**
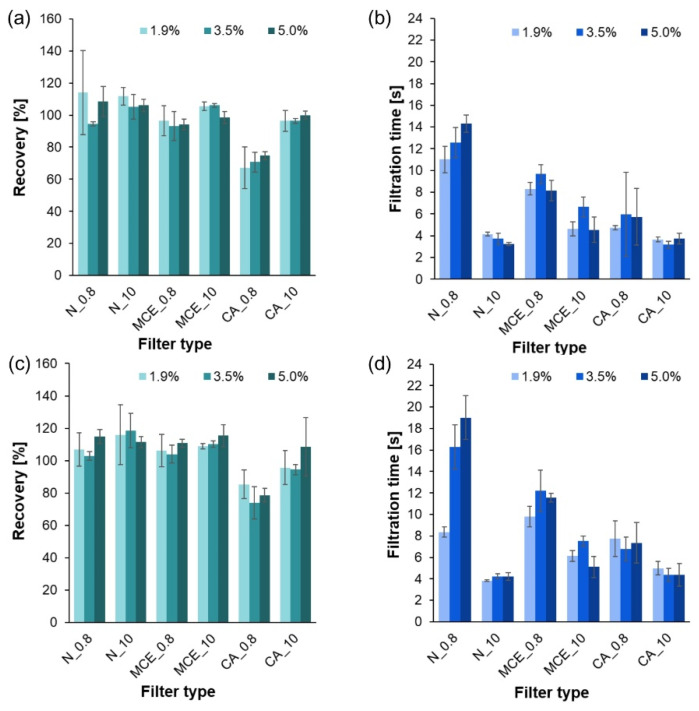
Results of the filtration process of systems treated with HCl solutions of 1.9, 3.5, and 5.0%: (**a**) recovery of PE in the system without sucrose; (**b**) filtration time of the system without sucrose; (**c**) recovery of PE in the system with sucrose; (**d**) filtration time of the system with sucrose.

**Figure A3:**
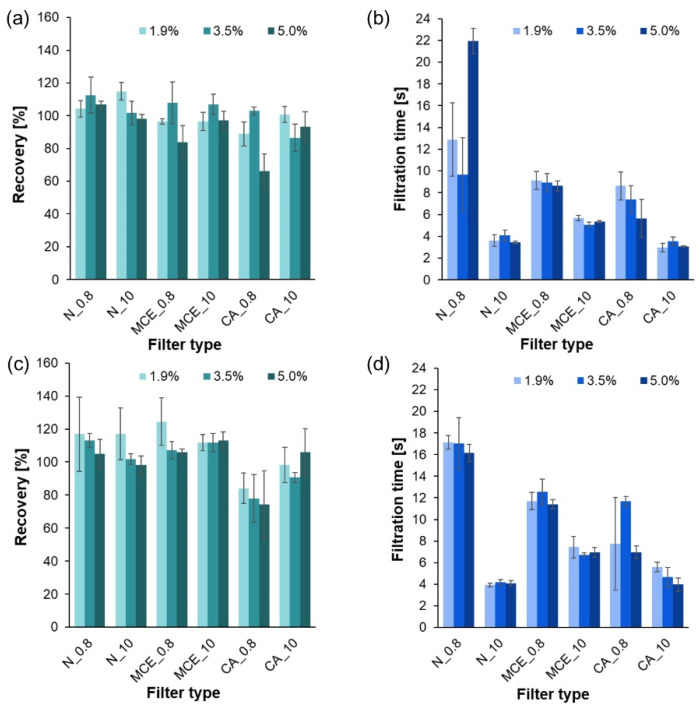
Results of the filtration process of systems treated with HNO_3_ solutions of 1.9, 3.5, and 5.0%: (**a**) recovery of PE in the system without sucrose; (**b**) filtration time of the system without sucrose; (**c**) recovery of PE in the system with sucrose; (**d**) filtration time of the system with sucrose.

To develop the MP isolation process, the filtration time was measured at each stage of the study, and the recovery of the method was assessed. The average results from all measurements are shown in Figure A1, Figure A2 and Figure A3 (Appendix A).

The statistical evaluation included two independent Shapiro–Wilk tests at the 95% significance level. The results for both the variables of mean filtration time (*p* = 0.000000) and mean recovery values (*p* = 0.000227) indicated that the collected data did not show a normal distribution (*p* < 0.05). Therefore, in further analysis, the Kruskal–Wallis test was applied at the 95% significance level.

The temperature dependence of filtration time and recovery was examined. The results were *p* = 0.9814 and *p* = 0.8789, respectively. Statistically significant differences were not recorded (*p* > 0.05). Thus, it was found that changing the temperature from 50 to 70 °C did not significantly affect the time or recovery.

The dependence of filtration time and recovery on the presence of sucrose in the studied systems was analyzed, obtaining *p* = 0.0348 and *p* = 0.0002, respectively. Statistically significant differences between the studied parameters were noted (*p* < 0.05). It was found that the presence of sucrose significantly affected filtration time (by lengthening it) and recovery (by increasing the obtained values as sucrose was deposited on MP particles).

Additional statistical analysis was also performed for systems differing in HCl or HNO_3_ concentrations. The concentrations of acids were not indicated to affect filtration time and recovery.

An analysis of recovery and filtration times in relation to filter type was also performed. The average recovery values and filtration times varied statistically significantly (*p* = 0.0000 < 0.05) depending on the filter selected. Post hoc tests were performed to show which groups differed. Multiple comparisons of mean ranks revealed that, in comparison with the other filters, the mean recovery varied and finally achieved the lowest value for a filter composed of CA with a pore size of 0.8 µm. Trials performed for the other filter types showed similar recoveries. In addition, the post hoc test results showed statistically significant differences in the filtration time of the solutions between the various filters. Filters with a pore diameter of 0.8 μm showed shorter filtration times than those with a diameter of 10 μm. Therefore, it was confirmed that the filtration time of the solutions was dependent on the pore diameter.

## 3. Discussion

Many conclusions about the impact of different factors on the filtration process and the properties of MPs can be drawn from the research presented in this work. It was found that neither the PE surface nor the efficiency of the filtration process had been impacted by increasing the process temperature to 70 °C. The particles’ appearance was significantly affected by the presence of sucrose, which led to their agglomeration. It is possible that a layer of sucrose, developed on the MPs’ surface, complicates surface analysis. Changing the procedure temperature to 70 °C reduced the amount of sucrose that covered the plastic particles, facilitating their identification. However, this change had no appreciable effect on the filtration time or process efficiency. In order to evaluate the surface modification, μ-Raman spectroscopy and SEM were used. The analysis of the test data demonstrated that temperature had no noticeable effect on the degradation of PE. However, the observed decrease in particle agglomeration at high temperatures may suggest that they affect the viscosity of sucrose. The kinetic energy of the particles in the solution increased as a result of the temperature rise. As smaller agglomerates might be simpler to filter, reducing agglomeration at higher temperatures might be advantageous.

In addition, the presence of sucrose affected recovery by increasing the obtained values. Thus, the hydrolysis of sucrose was necessary. The MPs’ surfaces became visible when acids were used to hydrolyze sucrose.

By comparing the degenerative effects of the acids used on MPs, it can be concluded that, in contrast to anaerobic acids, like HCl, acids with oxidizing properties, like HNO_3_, have a highly destructive effect on the surface of PE particles. On the PE surface, notable alterations were observed, even at a concentration of 1.9% HNO_3_, including a rise in the quantity of irregularities. The surface degradation intensified with increasing concentrations of this acid, leading to the formation of new functional groups (e.g., carboxyl groups), a shift in the position of the previously existing bands obtained for the reference polymers, and a change in their intensity. These findings are in accordance with those reported in the literature, which demonstrate that using hot HNO_3_ (80 °C for 2 h; 15.7 mol/L HNO_3_) damages a variety of polymers [25]. Polyethylene displayed surface alterations associated with the formation of new functional groups and a decrease in molecular weight after being exposed to such agents. Catarino et al. [33] (20 °C overnight followed by 60 °C for 2 h; 65%/14 mol/L HNO_3_), Karami et al. [31] (25 °C for 96 h; 69%/15.5 mol/L HNO_3_), Dehaut et al. [26] (20 °C overnight followed by 60 °C for 2 h; 65%/14 mol/L HNO_3_), and Claessens et al. [27] (20 °C overnight followed by 100 °C for 2 h; 95%/22.5 mol/L HNO_3_) reported similar outcomes. However, there was no surface yellowing on the tested PE. It can be presumed that, if the concentration of HNO_3_ was higher, this effect would be observed [26]. Consequently, it appears that using acids with oxidizing characteristics is improper for the majority of MPs analyses. This action could produce inaccurate analytical outcomes.

When testing samples with notable sucrose contents, 5% HCl was efficient after a brief exposure period and had no negative effects on PE. Similar conditions resulted in changes that HNO_3_ induced on the plastic’s surface. HCl produced the required separation, which facilitated further µ-Raman spectroscopy and polymer identification studies. As a result, MPs can be extracted using this acid with success. Respecting a particular digestion time is also essential when selecting a test protocol. Longer exposure times have the potential to improve the food matrix’s digestion efficiency, but they may also cause MPs to degrade in a way that has never been seen before.

The range of recoveries for systems containing sucrose digested with acids was 73.8 to 124.5%. The tests conducted are considered appropriate in the context of the European Parliament and Council’s (EU) guideline, which recommends that the recovery values be between 60 and 140% [34]. The reason for the differences obtained was not the digestion factors, but the type/quality of filter material used.

Several important findings were obtained in order to overcome the potential challenges related to the filters used for MPs’ isolation. Each filter was designed for a single use only, which imposed significant constraints on the experimental process. This limitation necessitated frequent filter replacements, thereby increasing the operational complexity and cost of the separation procedure. The applied filters did not clog during this research. However, it is noteworthy that higher concentrations of sucrose or larger volumes of solution could have led to these problems. In addition, the filters composed of CA exhibited greater brittleness compared with those made from a blend of cellulose esters and nylon. Cellulose acetate is a material in which the hydroxyl groups of cellulose are esterified with acetic acid, leading to a crystalline structure. Therefore, CA filters are brittle. Mixed cellulose esters are materials in which the hydroxyl groups of cellulose are esterified with different acids, creating a more amorphous structure. This increases the ability of the filters to deform. Nylon, on the other hand, is made of repeating units containing amide groups. These bonds are strong and stable, affording the filters high tensile strength and resistance to acids.

Depending on the kind of filter being used, there were variations in the filtration time and MP recovery across different filters, according to the analysis. In comparison with filters with larger pores (10 µm), those with smaller pore diameters (0.8 µm) exhibited longer filtration times. However, the possibility of using the technique in future structural studies must be considered when choosing the filter pore size and filtration time. In this field, µ-FTIR and µ-Raman spectroscopy are the most commonly used methods. Particles as small as 10 µm can be examined with the first, and as tiny as 1 µm can be investigated with the second. Consequently, it might be appropriate to use filters with a pore diameter of 10 µm in later µ-FTIR spectroscopy studies. As there are no statistically significant differences in the recovery values and filtration times between the described test results, all of the suggested pore size filters can be used with equal success. Filters with a pore size of 10 µm or smaller should not be used when using µ-Raman spectroscopy, as this will unnecessarily reduce the recovery value. As cellulose acetate filters can have recovery values lower by up to 20%, in this situation, 0.8 µm pore diameter nylon or mixed cellulose ester filters would be the best option.

In conclusion, this study’s variables, such as temperature, concentrations of HCl, HNO_3_, and the type of filter material, affected the process of separating MPs from solutions containing sucrose. The most effective technique for isolation is to use 5.0% HCl, which hydrolyzes sucrose in a solution heated to 70 °C for five minutes without altering the structure of MPs. For the accurate identification of MPs through chemical identification using spectroscopic techniques, the purification method chosen to remove organic matter from food samples is essential. This is crucial for particles that are several micrometers in size.

## 4. Materials and Methods

### 4.1. Chemicals and Materials

Throughout this study, all reagents met stringent analytical grade criteria. Hydrochloric acid (HCl) at a concentration of 37% was purchased from Supelco (Vienna, Austria). Nitric acid(V) (HNO_3_) at a concentration of 69% was obtained from Supelco (Darmstadt, Germany). Polyethylene (PE) particles (34–50 μm) were acquired from SIGMA-ALDRICH (St. Louis, MO, USA). Sucrose was purchased from Polski Cukier (Toruń, Poland). The production of ultrapure water involved the utilization of a Millipore Mili-Q system (Millipore Simplicity UV System, Millipore Company, Billerica, MA, USA). Before their use, all glassware underwent meticulous cleaning with water and detergent, followed by rinsing with distilled water and ultrapure water.

**Figure 4:**
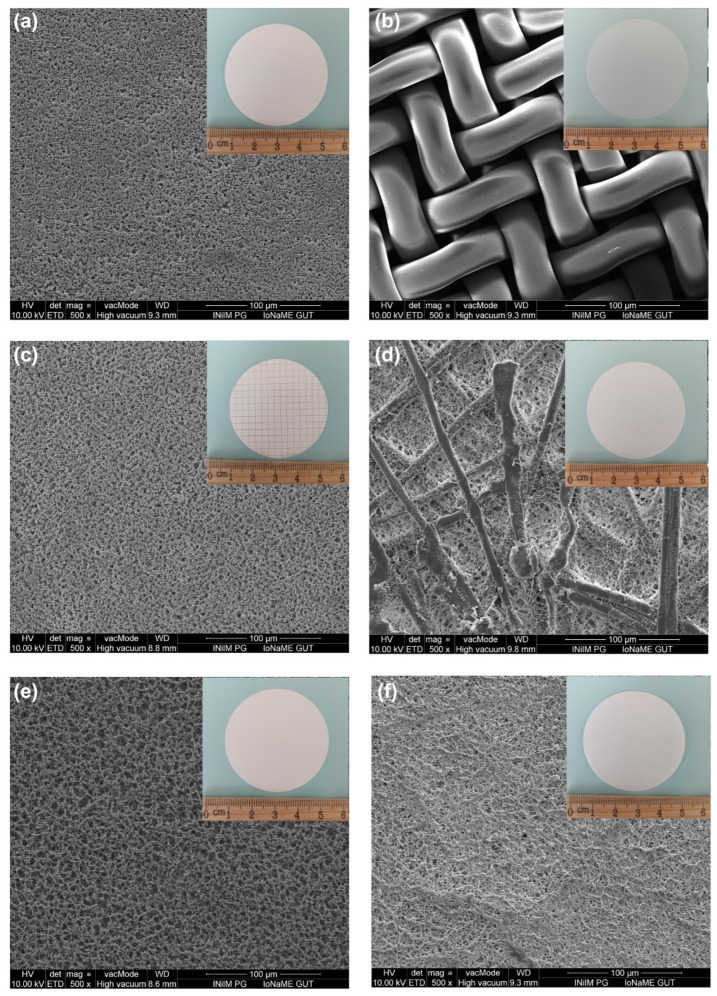
SEM images of (**a**) nylon filter of 0.8 µm, (**b**) nylon filter of 10 µm, (**c**) mixed cellulose ester filter of 0.8 µm, (**d**) mixed cellulose ester filter of 10 µm, (**e**) cellulose acetate filter of 0.8 µm, and (**f**) cellulose acetate filter of 10 µm.

The experiments included filters with different materials and pore sizes (Figure 4). These were nylon filters of 10 µm (Merck Millipore, Cork, Ireland), nylon filters of 0.8 µm (Chemland, Stargard, Poland), mixed cellulose ester filters of 10 µm (Qualimet, Ruda Śląska, Poland), mixed cellulose ester filters of 0.8 µm (CHEMLAND, Poland), cellulose acetate filters of 10 µm (Advantec, Tokio, Japan), and cellulose acetate filters of 0.8 µm (CHEMLAND, Poland).

### 4.2. Quality Control

To prevent the samples from being contaminated by the external environment during the examinations, extra precautions were taken. Throughout the experiment, researchers wore cotton lab coats because they were aware of the risks associated with clothing fibers. The laboratory’s conditions were also managed, and its doors and windows were closed. Strict hygiene regulations were also implemented for every piece of equipment used in this research. Every trace of plastic that would have compromised the quality of the outcome was removed by thorough cleaning.

Microplastics, after isolation on filters, were kept under strict control to guard against possible contamination. These filters (N_0.8, N_10, MCE_0.8, MCE_10, CA_0.8, and CA_10) were stored at room temperature in glass Petri plates inside a glass desiccator, protected from moisture and external plastic particles.

To ensure that the samples did not self-contaminate, control samples were prepared using ultrapure Mili-Q distilled water under all testing conditions. Control samples increased the dependability of the experimental results by acting as a point of reference.

### 4.3. Method Development

**Figure 5:**
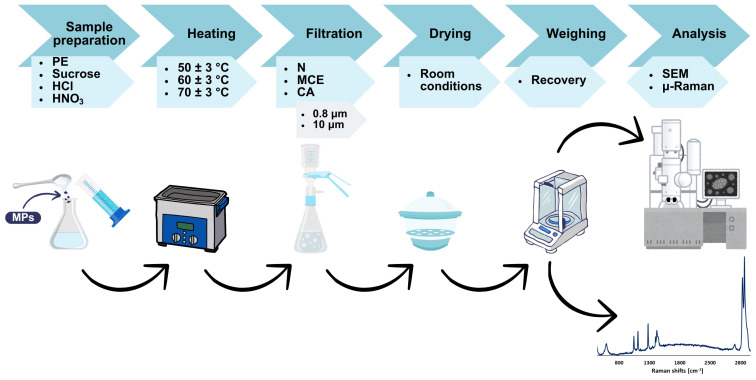
Overview of the experimental procedure.

The research methodology (Figure 5) is based on several test systems, the composition of which is shown in Table 1. Tests were conducted for various combinations of components, including ultrapure water, sucrose, HCl, HNO_3_, and MPs (PE 34–50 μm). The sample to control ambient contamination was ultrapure water (100 mL), while the reference sample was ultrapure water (100 mL) with MPs (0.01 ± 0.0009 g).

Three different temperatures were used for samples 1 and 2 (Table 1): 50 ± 3, 60 ± 3, and 70 ± 3 °C. For the other samples, only 70 ± 3 °C was used (this temperature does not degrade the MPs, but reduces the sucrose layer on the surface of the particles). All the systems shown in Table 1 and the reference samples were tested using each type of filter. The tests were performed in triplicate. Filtration time was studied for all systems, while for systems with PE (34–50 μm), recovery (R) and changes on the surface of MPs were also examined. The recovery was determined using the following formula:

(1) $$R=\frac{m_{3}-m_{2}}{m_{1}}\times 100\%$$

where m_1_ is the weight of MPs added to the sample (mg), m_2_ denotes the weight of the filter (mg); and m_3_ is the combined weight of the dried filter paper and MPs (mg).

### 4.4. Scanning Electron Microscopy

After filtration under different conditions, MPs on filters, previously coated with a 10 nm thick layer of gold, were checked by SEM (FEI Quanta FEG 250, FEI, Eindhoven, The Netherlands). Microplastics that were not subjected to any physicochemical factors served as the reference samples (raw). The filters were attached to the holder with carbon tape. The SEM analysis was performed in the high-vacuum mode using a secondary electron (SE) detector. The examination was conducted at an accelerating voltage of 10 kV with a working distance of c.a. 10 mm.

### 4.5. Micro-Raman Spectroscopy (µ-Raman)

After filtration, Raman spectra for polyethylene (PE) were collected using a confocal µ-Raman setup. This system consisted of a LabRam Aramis (Horiba Jobin Yvon) 460 mm spectrometer integrated with a confocal microscope. The excitation source comprised a diode-pumped solid-state laser generating green light at 532 nm. The measured spectral range extended from 400 to 3200 1/cm. A spectral resolution of 2 1/cm and a grating with 600 grooves/mm were used. The confocal aperture was adjusted to a width of 200 µm. Precise focus onto a 1–2 μm spot on the particle was accomplished using a 50× objective that had a numerical aperture of 0.5. Spectral data were collected through 20 scans lasting 2 s. Neutral-density filters ranging from 0 to 2 optical density were used to calibrate different signal intensities. The reference sample was raw PE (34–50 μm).

### 4.6. Statistical Analysis

The Shapiro–Wilk test was used to confirm the normality of the distribution of the data. Subsequent statistical analysis employed the Kruskal–Wallis test to explore relationships among the results obtained for various groups. Statistical significance was established for *p*-values less than 0.05. Subsequently, the post hoc (Dunn) test was applied to pinpoint groups exhibiting statistically significant differences. The analyses were conducted using Statistica 13.3 software from TIBCO Software Inc., based in Palo Alto, CA, USA.

## 5. Conclusions

The development of a method to isolate MPs from food products is essential for identifying the sources of these contaminants and removing plastic particles. This investigation successfully developed a method for the separation of MPs from sucrose-containing foods. It was indicated that 5% of HCl hydrolyzes sucrose in a solution heated to 70 °C for five minutes without degrading PE. This is the best way to break down sugar because it enables the identification of polymers later on. Therefore, recommendations for testing MPs from samples with a significant sucrose concentration could be based on the results of this investigation. We believe that our methodological approach, which standardizes isolation processes, will be helpful to researchers and practitioners investigating plastic particle contamination of food because it will make it easier to monitor MPs in sweetened products and implement appropriate countermeasures. Future research should explore the applicability of this methodology to other food matrices.

## Figures and Tables

**Table 1:** Compositions of the test systems.

No.	Test System	Amount of the Component			
		MPs	Sucrose	H_2_O	HCl/HNO_3_
1	H_2_O + MPs	10 ± 0.9 mg	–	100.0 ± 0.1 mL	–
2	H_2_O + Sucrose + MPs	10 ± 0.9 mg	10 ± 0.009 g	100.0 ± 0.1 mL	–
3	H_2_O + 1.9% HCl + MPs	10 ± 0.9 mg	–	95.0 ± 0.1 mL	5.0 ± 0.1 mL
4	H_2_O + 3.5% HCl + MPs	10 ± 0.9 mg	–	90.7 ± 0.1 mL	9.3 ± 0.1 mL
5	H_2_O + 5.0% HCl + MPs	10 ± 0.9 mg	–	86.4 ± 0.1 mL	13.6 ± 0.1 mL
6	H_2_O + 1.9% HNO_3_ + MPs	10 ± 0.9 mg	–	97.3 ± 0.1 mL	2.7 ± 0.1 mL
7	H_2_O + 3.5% HNO_3_ + MPs	10 ± 0.9 mg	–	95.0 ± 0.1 mL	5.0 ± 0.1 mL
8	H_2_O + 5.0% HNO_3_ + MPs	10 ± 0.9 mg	–	92.7 ± 0.1 mL	7.3 ± 0.1 mL
9	H_2_O + Sucrose + 1.9% HCl + MPs	10 ± 0.9 mg	10 ± 0.009 g	95.0 ± 0.1 mL	5.0 ± 0.1 mL
10	H_2_O + Sucrose + 3.5% HCl + MPs	10 ± 0.9 mg	10 ± 0.009 g	90.7 ± 0.1 mL	9.3 ± 0.1 mL
11	H_2_O + Sucrose + 5.0% HCl + MPs	10 ± 0.9 mg	10 ± 0.009 g	86.4 ± 0.1 mL	13.6 ± 0.1 mL
12	H_2_O + Sucrose + 1.9% HNO_3_ + MPs	10 ± 0.9 mg	10 ± 0.009 g	97.3 ± 0.1 mL	2.7 ± 0.1 mL
13	H_2_O + Sucrose + 3.5% HNO_3_ + MPs	10 ± 0.9 mg	10 ± 0.009 g	95.0 ± 0.1 mL	5.0 ± 0.1 mL
14	H_2_O + Sucrose + 5.0% HNO_3_ + MPs	10 ± 0.9 mg	10 ± 0.009 g	92.7 ± 0.1 mL	7.3 ± 0.1 mL

## Data Availability

The original contributions presented in the study are included in the article; further inquiries can be directed to the corresponding author.
